# Assessment of expenditure control and prescriptive appropriateness of biological drugs in autoimmune diseases and chronic inflammatory bowel disease

**DOI:** 10.3389/fphar.2013.00031

**Published:** 2013-03-22

**Authors:** Maurizio Capuozzo, Eduardo Nava, Stefania Cascone, Claudia Cinque, Roberta Marra, Alessandro Ottaiano, Corinne Scognamiglio, Emilia Palumbo

**Affiliations:** ^1^Department of Pharmacy at the ASL Naples 3Ercolano, Italy; ^2^Department of Pharmacy at the ASL Naples 3Nola, Italy; ^3^Pharmacist at the ASL Naples 1 CenterNaples, Italy; ^4^Department of Colorectal Oncology at the National Cancer Institute, “G. Pascale” FoundationNaples, Italy; ^5^Professional Nurse, Department of Pharmacy at the ASL Naples 3Ercolano, Italy

## Introduction

Therapies with biological drugs represent a very important pharmacological resource available to the rheumatologist and all those patients who see compromised the quality of their life because of a lack of response to traditional therapies. The Italian Society of Rheumatology (SIR) is strongly committed to ensure patient access to the best treatment available, paying attention to limited economic resources. The new Guidelines of the SIR go in this direction. With the term “biological drugs” are considered all those new generation drugs designed to act only on a single structure (which may be a protein, a receptor, or even a DNA sequence) by increasing the efficacy of the therapy and by reducing, at the same time, the undesired effects. Today there are biological drugs available against autoimmune diseases (for example, lupus, rheumatoid arthritis, psoriasis), chronic inflammatory diseases of the intestine (such as Crohn's disease and ulcerative colitis), and some types of cancer. This study takes its cue from Commissary Decree n. 26 of 14 March 2012 (region of Campania, Italy), on the appropriateness of biologics used to treat psoriasis and psoriatic arthritis, rheumatoid arthritis, ankylosing spondylitis, and Chronic inflammatory diseases of the intestinal tract both in adult and pediatric patients, excluding therefore biological drugs prescribed for tumor pathologies. The objective of biologics is to reach the cells or diseased structures, by acting directly on these without damaging healthy cells. To achieve that goal it was thought to use the defenses of our organism, namely antibodies, modifying them in such a way as to make them capable to recognize as aggressors structures diseased or proteins involved in the pathological process. As in the case of the studied drugs to treat autoimmune diseases, such as rheumatoid arthritis, that are programmed for “attacking” the pro-inflammatory cytokines released by inflamed cells, so as to block the inflammation. The purpose of this study is to compare the requirements of biological drugs for the mentioned diseases, monitor accurately spending of these drugs with a high cost and check constantly the appropriateness prescriptive with the adoption of the regional tab for the prescription from the centers of reference having the requirements of the law.

## Materials and methods

In the Pharmacy of the District of Herculaneum (Naples, Italy) we have activated by April 2012, an archive H of biological drugs purchased and dispensed. All the regional cards, comprising the biological drugs prescribed, received during the period April 2012–September 2012 were examined. These cards are the pre-printed forms each for a particular therapeutic indication for which the biologic drug is prescribed. It consists of four sections. The first section shows the data of the reference center and the prescribing physician, the second section of the card shows the generality of the patient, the third section includes the diagnosis and the biologic drug prescribed with specified the dosage and the mode of administration while the fourth section is for recording the delivery of the drug by the pharmaceutical service of the ASL of residence of the assisted. This card is valid for 3 months and also contains a small statement of acquisition by the prescribing physician, the informed consent of the patient. The archive, on excel sheet, has allowed us to have sure data and immediate on the number of therapies with each drug monitored and has been created so that each patient is recorded and catalogued on the basis of biologic drug prescribed, dosage, and expenditure calculated for single vial delivered.

## Results

During the reporting period April 2012–September 2012 were treated a total of 79 patients with autoimmune diseases or chronic inflammatory bowel disease. The biologics prescribed were Etanercept fl 50 mg, Adalimumab fl 40 mg, Infliximab fl 100 mg, Tocilizumab fl 80 mg–200 mg–400 mg, Ustekimumab fl 90 mg, Certolizumab fl 200 mg, Abatacept fl 250 mg. Specifically, the data obtained were: 41 patients treated with etanercept fl 50 mg and delivered 396 vials in all for a total expenditure of about €94,799. Of the 41 patients treated with etanercept fl 50 mg, 20 were suffering from psoriatic arthritis, 12 from rheumatoid arthritis and 9 suffering from psoriasis; in addition, 20 patients were treated with Adalimumab fl 40 mg to which were delivered 110 vials for a total expenditure of about €51,412. Of the 20 patients treated with Adalimumab fl 40 mg, 7 were suffering from rheumatoid arthritis, 7 psoriatic arthritis, 4 psoriasis and 2 suffering from Crohn's disease; were also 4 patients treated with Infliximab fl 100 mg to which were delivered 28 vials in all for a total expenditure of about €13,055. Of the 4 patients treated with Infliximab fl 100 mg, 3 were with psoriatic arthritis and 1 suffering from psoriasis; in addition, 5 patients were treated with Tocilizumab fl 80 mg, 200 mg, and 400 mg, which were delivered 53 vials in all for a total expenditure of about €12,863 all suffering from rheumatoid arthritis; in addition, 2 patients with both psoriasis were treated with Ustekimumab fl 90 mg to which were delivered 2 vials in everything for a total expenditure of about €5686; other 2 patients with both rheumatoid arthritis were treated with Certolizumab fl 200 mg to which were delivered 10 vials for a total expenditure of about €3415; finally, 5 patients were treated with Abatacept fl 250 mg to which were dispensed in all 39 vials for a total expenditure of about €12,600 and all suffering from rheumatoid arthritis. The pharmaceutical spending total, incurred to acquire biological drugs to assisted the District of Herculaneum, was approximately €197,000 and the diseases treated were rheumatoid arthritis, psoriatic arthritis, psoriasis and Crohn's disease for a total of 79 patients treated on a population of 57,000 inhabitants in the territory of Herculaneum (Naples, Italy). Rheumatoid arthritis has resulted in the pathology more treated with 31 patients, 30 patients instead with psoriatic arthritis, 16 affected by psoriasis and 2 suffering from Crohn's disease. The most prescribed drug was Etanercept fl 50 mg which has had an impact on total expenditure for the approximately 48%, while Adalimumab fl 40 mg has had an impact on total expenditure for the 26% approximately. The Infliximab fl, Tocilizumab fl, and Abatacept fl have contributed each for the 6.5% while Ustekimumab and Certolizumab have participated on total expenditure in a manner quite negligible around 2–3%.

## Discussion

The pharmaceutical service in the District of Herculaneum did spent in the 6-month period from April 2012 to September 2012, in all about €2,000,000 for the pharmaceutical care of all the sick afferent to the District. If one considers that it is only for the purchase of biological drugs, for the aforementioned pathologies, were spent approximately €197,000 or almost 10% of the total, we can actually conclude by saying that the biological drugs have a high impact on pharmaceutical spending overall, therefore, a careful monitoring is really necessary for the purpose of improving the quality of care to obtain a more efficient management of the resources available. The National Guidelines and International, based on scientific evidence, define precise criteria to minimize the variability in clinical practice, supporting the rheumatologists, in their daily work, in finding the best treatment that takes into account the specificity of the individual patient, avoiding delays in the choice of therapy most suited and optimizing especially the use of drugs biologists with obvious repercussions on health spending. The objective is to identify patients eligible for treatment with biological drugs, with the aim to identify those people who, by undergoing biological therapy, may actually benefit from them. The introduction of biological drugs in clinical practice has certainly represented an absolute revolution in rheumatology, both for the innovative design of aim of the treatment on specific targets fundamental in the pathogenesis of the disease, and both for the undoubted efficacy demonstrated by these drugs against the traditional therapies. The extensive use of this therapeutic approach is however, still limited by several factors, such as the lack of information about the security in the long term, the need for a monitoring of patients more attentive and narrow, the limitations in the selection of subject treatable set by the criteria of inclusion and exclusion from treatment and, no less important, the absolute cost of biological medicines, as demonstrated in this study. For this therapy with biological and still reserved for the prescription of rheumatologic centers of excellence, through a careful selection of cases in order to identify the patients most suited to this therapeutic regimen. It is obviously desirable that in the coming years a better knowledge of long-term effects of biological drugs and a better economic planning of their use can increase progressively the use, extended to cover a proportion of patients increasingly wide.

